# Illinois State Veterinary Medical Association

**Published:** 1897-01

**Authors:** Albert Babb

**Affiliations:** Secretary


### SOCIETY PROCEEDINGS.

### ILLINOIS STATE VETERINARY MEDICAL ASSOCIATION.

The thirteenth annual meeting was held at the Sherman House, Chicago, November 17th and 18th, and was called to order by President M. R. Trumbower at 3 P.M. On roll-call the following members responded: Drs. A. G. Alverson, Albert Babb, A. H. Baker, S. S. Baker, F. L. Brown, John Casewell, L. Campbell, E. S. Frye, Robert Gysel, T. J. Gunning, W. C. Hanawalt, H. G. Hoover, Joseph Hughes, C. W. Johnson, J. H. Judson, O. J. Lanigan, W. J. Martin, C. A. Pierce, E. L. Quitman, J. T. Ryan, C. E. Sayre, J. L. Siegrosser, Olof Schwarzkopf, J. L. Tyler, M. R. Trumbower, W. F. Weise, M. Wilson, and R. G. Walker.

The President then made his annual address, which was pregnant with facts regarding the present status of the profession in this State. Dr. E. S. Frye then read his paper, "Inversion of the Uterus," which was liberally discussed, after which came the production of Dr. Charles W. Johnson on "Parturient Apoplexy," which occasioned the usual protracted discussion of this perplexing disease.

On application the following gentlemen were admitted to membership: Drs. J. F. Black, A. E. Flowers, James Henderson, L. A. Merillat, George McEvers, James Robertson, all of Chicago, together with Drs. Harri H. Dell, of Sullivan, Ill.; G. A. Lytle, of Barrington, Ill.; G. G. Ratz, of Red Bud, Ill.; and W. H. Welch, of Lexington, Ill.

At the evening session the Society listened to the meritorious paper of Dr. E. L. Quitman on "Therapeutics." A spirited and lengthy discussion followed. A vote of thanks was extended to the writer. Society adjourned until the next day.

The Society was called to order at 10 A.M., November 18th, with the President in the chair. Dr. W. C. Hanawalt read his essay on "Therapeutics of Salicylate of Sodium," which was liberally discussed. Next came Dr. W. J. Martin with his article on "Fistulous Withers," which brought forth many practical suggestions. Adjournment for dinner then followed.

Society returned to business at 3 P.M., and at once listened to Dr. W. F. Weise's paper, "Infectious Ophthalmia of Cattle." Several members then related their experiences with this disorder. Then came Dr. James Robertson's production, "The Relation of a Veterinarian to the Horseshoer." It was a sound practical article on a very important subject, and was duly appreciated.

A recess of fifteen minutes was then taken for a general talk on the matter of legislation, after which the Treasurer, Dr. R. G. Walker, made his report, showing a small balance to the credit of the Association.

Election of officers was next in order, and the following were chosen: President, M. R. Trumbower, of Sterling, re-elected; Vice-President, A. G. Alverson, of Bloomington; Secretary, Albert Babb, of Springfield, re-elected; Treasurer, R. G. Walker, of Chicago, re-elected. The present Board of Censors was continued another year. The President appointed the following Committee on Programme: Drs. A. H. Baker, J. F. Pease and J. L. Siegrosser.

On motion, the chairman appointed Drs. S. S. Baker, M. Wilson, T. J. Gunning, J. L. Tyler, and Albert Babb as a Committee on Legislation, with power to act. All were determined to work for a bill in the coming session of the Legislature, and the Society assessed each member ten dollars to make up the requisite fund for expenses of the committee. Dr. Drinkwater, of Iowa, was a welcome visitor. Col. Hamilton was also present and gave some timely suggestions in regard to legislation.

On motion, the Society adjourned to meet at the call of the President at Bloomington some time in February. ALBERT BABB, A.B., M.D.C.,
Secretary.

